# Editorial: Infectious diseases, microbial ecology, and antimicrobial resistance dynamics in food animals

**DOI:** 10.3389/fvets.2023.1266980

**Published:** 2023-08-08

**Authors:** Sahar A. Kandeel, Ameer A. Megahed

**Affiliations:** ^1^Department of Animal Medicine (Infectious Diseases), College of Veterinary Medicine, Benha University, Kalyobiya, Egypt; ^2^Department of Large Animal Clinical Sciences, College of Veterinary Medicine, University of Florida, Gainesville, FL, United States; ^3^Department of Animal Medicine (Internal Medicine), College of Veterinary Medicine, Benha University, Kalyobiya, Egypt

**Keywords:** microbial ecology, antimicrobial resistance, food animals, environment-microbe interaction, host-microbe interaction

Infectious diseases of animals are a highly interdisciplinary subject that requires studying multiple disciplines, particularly biological understanding of the pathogen, a statistical description of the available data, and mathematical models for prediction. The dynamics of infectious diseases are complex and can be difficult to predict. In some cases, infectious diseases can spread rapidly through a herd or flock, leading to significant losses. In other cases, the disease may be more localized but still poses a risk to animal health and food safety. By understanding the dynamics of these diseases, we can take steps to prevent their spread and implement appropriate control measures to protect the health of food animals and humans. However, the dynamics of infectious diseases in food animals are constantly changing as new pathogens emerge and new strains of existing pathogens become resistant to antibiotics (1).

The widespread of infectious diseases has redirected scientists' attention to comprehensively studying the host-pathogen interaction in order to create innovative preventative and management measures to combat these diseases. Recent advances in sequencing technologies have revealed new insights into the composition and function of the microbiome across a wide range of hosts and environments. As a result, the study of animal microbiomes has become a growing area of research and is now recognized as an important driver of animal health and productivity (2, 3). Therefore, understanding the complexity of animal microbiomes and their interactions with the host is essential for developing strategies to improve animal health and production while reducing disease incidence. Microbiota harbored at different anatomic sites across the animal body and differ in microbial composition and function (4). Several factors significantly impact host-microbe interactions, such as host physiology, environmental exposures, etc. (4). Over the past decade, understanding microbial ecology fortifies the ability to diagnose or predict disease risk in the absence of a complete understanding of causal mechanisms. Furthermore, several studies have reported that microbiota is an important reservoir for antimicrobial resistance genes (ARGs).

Uncontrolled use of antimicrobials can disrupt colonization resistance, which leads to colonization by pathogens, increased load, dissemination of ARGs, and, eventually, clinically significant infections (5). However, the intersection of infectious diseases and antimicrobial resistance represents an important knowledge gap. The dynamics of AMR in food animals is a complex process that is influenced by a number of factors, including the use of antimicrobials, the microbial ecology of food animals, and the environment. Antimicrobial resistance can evolve through a variety of mechanisms, including mutation and gene transfer. Furthermore, once a bacterium becomes resistant to an antimicrobial, it can pass this resistance to other bacteria. Antimicrobial-resistant bacterial infections now account for many emerging infectious diseases worldwide (6, 7). Additionally, the use of antibiotics is known to affect the dynamics of infectious diseases in food animals. Furthermore, the global consumption of antimicrobials is expected to increase by 67%, between 2010 and 2030 (8). Therefore, it is important to stay up to date on the latest information so that appropriate measures can be taken to protect animal health and food safety.

This Research Topic aims to focus on discovery and innovation in various aspects related to infectious diseases, microbial ecology, and antimicrobial resistance dynamics in food animals and its role in enhancing animal health and profitability. In this Research Topic, there are five published papers covering the mentioned aims.

The environment can provide a reservoir for pathogens, as well as a means for pathogens to spread from one animal to another. However, there are some general principles that can be applied to understand how these diseases spread including the number of susceptible animals in a population, the contact rate between animals, and the virulence of the pathogen. In this Research Topic, Huma et al., described the phenotypic and molecular characterization of bovine mastitis milk-origin bacteria and the linkage of intramammary infection with milk quality. This study showed a significant negative correlation of SCC with milk components with the presence of various toxic genes in different milk culture isolated bacteria. In conclusion, mastitis-affected milk contains numerous pathogenic bacteria, toxins, and reduced milk quality, which is unfit for human consumption.

The intensification of modern animal husbandry, especially in the Western world, has led to an increase in infectious diseases and the emergence of multifactorial diseases (9). Dias and De Buck studied alternative less-invasive sampling techniques (swabs and fine-needle aspiration) in detecting and quantifying seven Digital Dermatitis (DD)-associated bacteria. Their results revealed that the alternative less-invasive sampling techniques were equal in their capacity to tissue biopsies to detect Treponema species with a more limited agreement for the other anaerobes. Additionally, bacterial numbers were higher in swabs and lower in aspirates compared to biopsies, therefore, aspirates were less suitable for bacterial quantification in DD lesions compared to the other methods.

Although antibiotics are often used to treat infections, they can also contribute to developing antimicrobial resistance (AMR) which is considered a growing global health threat. These resistant strains can then spread to other animals and to humans, making it more difficult to treat infections. Martínez-Vázquez et al., evaluated the multidrug resistance profile of *E.coli* strains isolated from bovine feces and carcass samples in Northeast Mexico. The results showed that a multidrug-resistant phenotype was found in 72.7% of the tested strains with 94.8% of isolated *E.coli* showing resistance to at least one antimicrobial, mainly ampicillin, cephalothin, and tetracyclines. These results indicate that bovine-origin food products in this area can be a source of multiresistant *E.coli* strains for the environment and exposure for consumers. Similarly, Massé et al., studied the prevalence of antimicrobial resistance and characteristics of *E.coli* isolates from fecal and manure pit samples on dairy farms in the province of Québec, Canada, and found that AMR is low for the highest priority critically important antimicrobials for humans and the highest levels of AMR were to tetracycline, sulfisoxazole, and streptomycin. However pre-weaned calves carried higher levels of AMR in commensal fecal *E.coli* than cows and manure pit systems on dairy farms with a high prevalence of extended-spectrum β-lactamase (ESBL) *E.coli* which is a possible contamination of the environment and raises public health concern. A number of things can be done to help prevent the spread of AMR, including using antibiotics judiciously, improving sanitation practices, and educating consumers about the importance of food safety. There is a need for more research on AMR in food animals. This research will help us to better understand the problem and to develop effective solutions.

The immune system plays a vital role as the body's natural defense system, actively safeguarding against infections which are considered a significant preventive measure, effectively mitigating the spread of infectious diseases among animals. In this context, trace minerals emerge as valuable allies in supporting the immune system's effectiveness, making it more adept at warding off harmful pathogens (10). Data from Kandeel et al., summarized camels' enzymes and proteins, which allow them to thrive under varied harsh environmental situations. This study revealed the existence of protein-coding and fast-developing genes that govern a variety of metabolic responses. Moreover, camels developed several paths to get optimum levels of trace elements which have key importance in their body for normal regulation of metabolic events.

## Author contributions

SK: Writing—original draft, Writing—review and editing. AM: Writing—review and editing.
